# Supplementary material for: A binary system in the S cluster close to the supermassive black hole Sagittarius A*
Source: Nat Commun. 2024 Dec 17;15:10608. doi: 10.1038/s41467-024-54748-3 (PMC11652627; doi:10.1038/s41467-024-54748-3)
Supplement: Supplementary file 1 — Supplementary Information [file 41467_2024_54748_MOESM1_ESM.pdf]

Supplementary Information for

A binary system in the S cluster close to the supermassive black hole Sagittarius A*

Florian Peißker[1*], Michal Zajaček[2,1], Lucas Labadie[1], Emma Bordier[1], Andreas Eckart[1,3], Maria Melamed[1], Vladimír Karas[4]

[1*]1.Physikalisches Institut, Universität zu Köln, Zülpicher Str. 77, Cologne, 50937, Germany.
[2]Department of Theoretical Physics and Astrophysics, Masaryk University, Kotlářská 2, Brno, 61137, Czech Republic.
[3]Max-Plank-Institut für Radioastronomie, Max-Planck-Gesellschaft, Auf dem Hügel 69, Bonn, 53121, Germany.
[4]Astronomical Institute, Czech Academy of Sciences, Boční II 1401, Prague, 141 00, Czech Republic.

*Corresponding author(s). E-mail(s): peissker@ph1.uni-koeln.de;

Supplementary Methods

Image sharpener

Filtering techniques are a common tool to enhance the amount of information that can be extracted from astrophysical data. For example, the Keplerian approximation presented in [1] for the B2V star S2/S-02 is based on high-pass filtered data observed with the NTT telescope. In addition, the comprehensive list of S-stars provided by [2] used a high-pass filtering technique. For the photometric analysis presented in this work, we applied a robust image sharpener to the data to filter the high spatial frequencies associated with noise and destructive PSF features that hinder the detection of faint sources. For this, we apply a PSF-sized smoothing kernel to the data I_{data} to obtain a blurred version I_{smooth} of the input file. By subtracting I_{smooth} from I_{data}, we get the sharpened version of I_{data}. For visual improvements, the final sharpened output can be convolved again with a Gaussian kernel, as was done for the multi-wavelength detection of D9 presented in this work. The astrometric and photometric robustness of this method is demonstrated in [3], where the impact of the image sharpener on all stars observed in the NSC has been analyzed in detail using NACO data. For this work, we use the H+K SINFONI data and compare the filtered and non-filtered data. We apply a 6 pixel (75 mas) Gaussian to the data displayed in the finding chart of D9 and detect in total 53 sources in the non-filtered H and K band data. In the filtered data, we detect about 20% more sources as a result of the reduced impact of the PSF wings of the stars, which is expected if we compare the total number of S stars reported in [4] and [5]. Eisenhauer et al. used continuum data and reported about 30 S stars. In comparison, Ali et al. used high-pass filtered data and showed approximately 20% more stars inside the inner 40 mpc. As a qualitative measure, we compare the

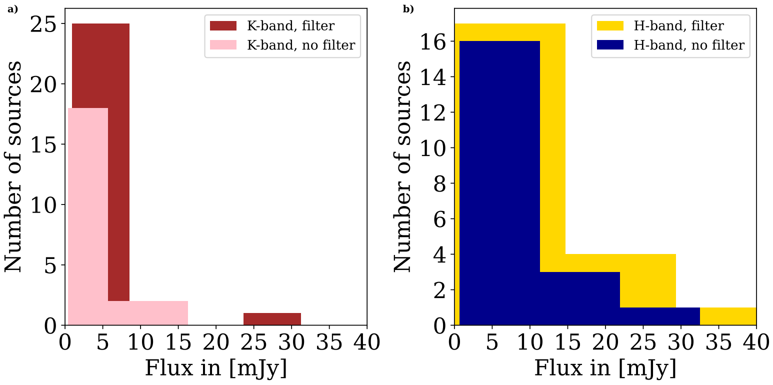

Supplementary Figure 1 Robustness of the filtering technique used in this work. We compare the flux of single S-stars in the H- and K-band for filtered and non-filtered data. In subplot (a), we show the K band, whereas subplot (b) represents the H band data. Due to the nature of the image sharpener, we detect about 20% more individual sources in the filtered data because of the reduced impact of the PSF wings. The bin size in the histograms is half of the square root of the number of sources.

median filtered and non-filtered flux density values for the related infrared bands (Fig. 1). We list the resulting flux density values in Table 1 and find deviations between the filtered and non-filtered data in the range of about $2-3\%$. Taking into account

	H band [mJy]	K band [mJy]
Non-filtered	5.49	3.36
Filtered	5.78	3.54
Standard deviation	0.14	0.09

Supplementary Table 1 Median flux density values for the S stars. The standard deviation is estimated from the flux values.

the expected deviations that are associated with observations of the Galactic center of approximately $10-20\%$ [3, 6, 7], the application of an image sharpener does not alter the photometric results analyzed and discussed in this work. Furthermore, the derived flux uncertainties are similar to the estimates used and discussed in [8].

Photometric analysis

For the photometric analysis of D9, we use the B2V star S2 as the reference source with related dereddened magnitudes of m_H =15.9±0.1, m_K =14.1±0.1, m_L =12.6±0.7 [9]. In the continuum data, the D9 system faces challenging confusion effects from nearby stars, such as S2, S12, and S39, so we focus on later epochs to detect the isolated H, K, and L band emission associated with the Doppler-shifted Brγ line emission. To minimize the impact of overlapping PSF wings in the crowded S cluster, we subtract a Gaussian-smoothed version I_{smooth} of the input data I_{in}. This post-processing technique is suitable for a wide range of astrophysical objects [10] and is characterized by its robustness with respect to image information conservation [3]. We use I_{in}-I_{smooth} to obtain I_{out} which is shown in the finding chart of D9 that represents a combination of H+K SINFONI data and NIRC2 (L band) observations carried out in 2019. For every data set, we identify a continuum source at the position of the NIR Doppler-shifted Brγ emission line and recover the corresponding flux information. We estimate 0.8±0.1 mJy, 0.3±0.1 mJy, and 0.4±0.1 mJy in the H, K, and L band, respectively. The respective dereddened NIR and MIR magnitude values are m_H = 19.92, m_K = 18.17, and m_L = 15.92. From the H-K and K-L colors, we compare the D9 system with other known objects in the S cluster and the NSC (Figure 2). While S stars such as S1, S2, and S4 can be classified as B0-B3 stars [11] which is reflected in their related H-K and K-L colors, we find photometric similarities between D9 and two close-by sources D2 (alternatively G3) and D23. All the three sources are located very close to the one-component linear fit (solid black line in Fig. 2) emphasizing their multi-wavelength nature. The location of D9 in the color-color diagram suggests a source that already shows characteristics of a main sequence star with a weak dust component.

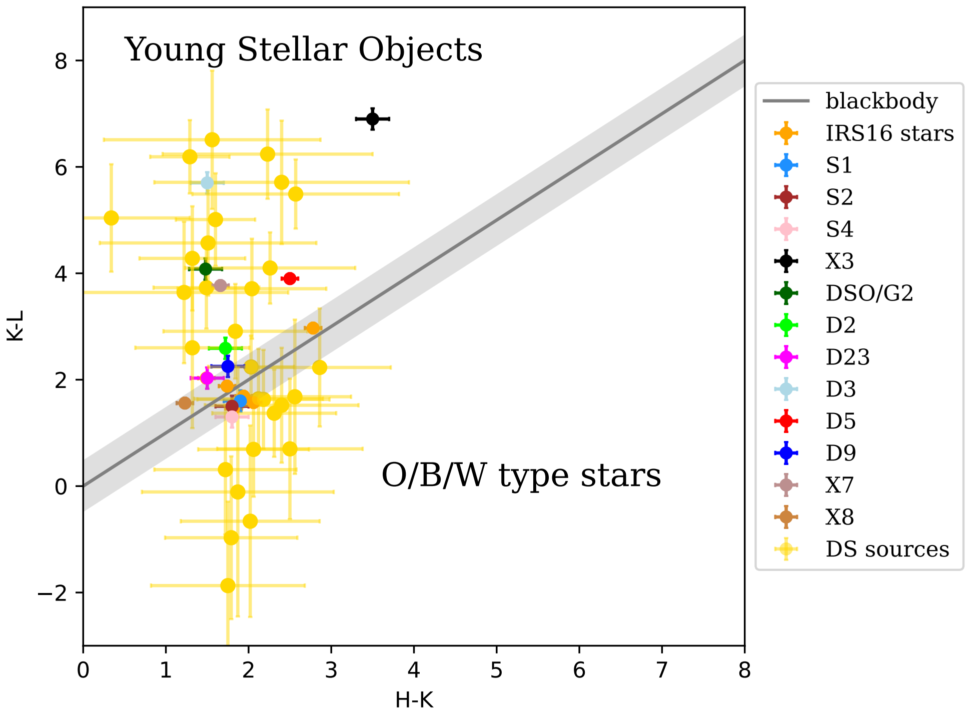

Supplementary Figure 2 Color-color diagram of prominent sources of the NSC. In this figure, the linear gray fit represents a blackbody. The young B-stars S1, S2, and S4 are below this linear blackbody fit. All G-objects are above this fit implying a different nature as the main sequence stars of the S cluster. For comparison, the yellow data points are taken from [3] and show the dusty sources in the IRS 13 cluster.

Extinction

Although the extinction of the reference source S2 was covered in detail in [12] and [13], we explore the impact of different values on the stellar parameters of the D9 system. The extinction law derived from [14] and the related magnitudes resulted in the flux density values for the SED presented in this work. As the magnitudes and, consequently, the flux density values estimated for D9 are extinction corrected, a range of dust densities is introduced into the model, which in turn affects the degree of reddening. These various dust densities can be described with visual extinction A_V, which is set to 0 in the SED fit presented in this article (because the magnitudes of the reference star are already extinction corrected). To investigate the impact of variable reddening, we use A_{V1}=1 and A_{V2}=2, which are 2-4 times larger than the usual uncertainties for optical extinction [14]. We list all the results in Table 2, where we implemented different optical extinction values. As is evident from Table 2, the different extinction values do not significantly impact the stellar radius or the temperature. Since both values are directly related to the position of D9 on the isochrones shown in

A_V	Radius in $[R_\odot]$	Temperature in log(T[K])	M_{Disk} in $[M_\odot]$	Envelope size in [AU]
31	2.00 ± 0.13	4.07 ± 0.05	1.61×10^{-6}	50.43
32	2.12 ± 0.33	4.10 ± 0.09	7.37×10^{-5}	85.84
33	2.22 ± 0.42	4.12 ± 0.08	2.61×10^{-3}	136.20

Supplementary Table 2 Comparison of the impact of different extinction values. For all three inspected extinction values, the stellar radius and temperature of D9 are comparable inside the uncertainties calculated by the radiative transfer model.

Fig. 4 in the main text, we find a stellar age estimate of $2.7^{+1.9}_{-0.3} \times 10^6$ yr. In deriving the disk mass and envelope size of the system using HYPERION, we find values that differ by a factor of one to three (Table 2). Although the small envelope (compared to, e.g., X3 [15]) is in line with the mass of the shallow circumbinary disk for all the three extinction values, we want to explore a possible impact on the expected thermal emission in the L band. For this, we will use the empirical model of [16] to calculate the dust sublimation radius r_{sub} and the outer radius of the envelope r_{out} that still contributes to the detected emission. Assuming a temperature of approximately 1000 K where dust photoevaporates due to stellar winds, we find a sublimation radius of $r_{sub} = r_{star}(T_{dust}/T_{star})^{-2.085} = 1.2$ AU. Due to the presence of the secondary, r_{sub} = 1.2 AU can be interpreted as a lower limit because we find a matching effective orbital radius of r_{eff} of approximately 1.2 AU. Presumably, the secondary has cleared the inner dust region of the primary as it has been observed for PDS 70 [17] or HD 104237 [18]. For an upper limit of the envelope size, we use the usual detection limit for dust sources in the GC of around 200 K [19]. We estimate an approximate minimum envelope radius of 50 AU and conclude that the L band emission for all the three extinction values should be observable on a comparable level. Due to dust shielding, the largest envelope listed in Table 2 will have low temperatures below the NIRC2 detection limit in the L band of 0.04 mJy. The detection limit for colder regions further away from the central star estimated in this work is in agreement with the analysis of [20]. An additional effect of different extinction values on the stellar parameters is the disk mass M_{Disk}. However, we should note that the impact on the radius and temperature of D9 is limited, as shown in Table 2. Consequently, we do not expect a significant variation in the mass M_{D9a} of the primary. This is not surprising since a correlation between M_{Disk} and stellar mass has not been observed so far [21]. Overall, we find that the extinction used in this work is robust against possible variations on small scales [12, 22].

Instrumental effects

As mentioned, the data are corrected for the variation of the barycenter of the Earth. However, we exclude the possibility of a false detection due to instrumental effects by investigating the close-by source D23. We use the same data set that resulted in the detection of the binary system D9 for the analysis of the Doppler-shifted Brγ emission line of D23. Due to the confusion-free detection of D23 between 2005 and 2019, as shown in Fig 3, we conclude that instrumental effects do not cause the periodic RV pattern. Furthermore, the two ERIS data points observed in 2022 show the same pattern as observed with SINFONI. Even if both instruments were to generate a

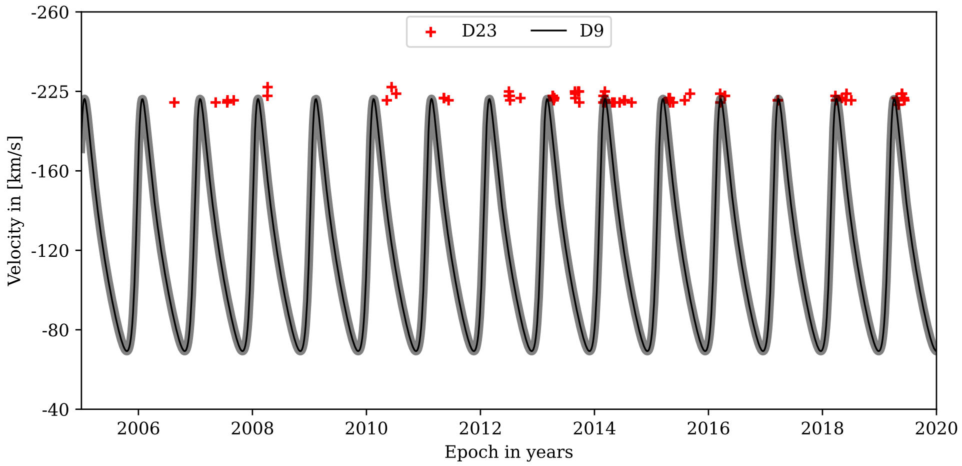

Supplementary Figure 3 Blue-shifted LOS velocity of D23. The close-by source with a velocity of -220.16 ± 2.47 km/s shows no signs of a periodic signal, which excludes an artificial origin of the RV pattern of D9. The uncertainty indicated reflects the standard deviation of the observed LOS velocities. The gray pattern shows the RV model displayed in the periodic pattern of D9 and demonstrates the magnitude of the LOS variability of D9 as compared to D23.

periodic pattern artificially, it is implausible that the magnitudes of these signals would be in agreement. We add that no periodicity is reported for S2 (S0-2) by examining the Brγ absorption line with SINFONI [23], strengthening our earlier argument.

Data quality

For the validity of the findings, we explore the quality of the data used to derive the periodic signal of the binary and listed in Tables 7-9 by classifying the observations in medium and good. For this classification, we fit a Gaussian to the K band continuum emission of S2 (S0-2) and compare the Full Width Half Maximum (FWHM) of each data set. A high quality is represented by diffraction-limited values close to 6 pixel (75 mas), whereas medium data are close to 7 pixel (87.5 mas). Usually, higher values forbid confusion-free detection of individual S stars in the cluster. In addition to this quality control, we measure the SNR level to explore the influence of quantities beyond the initial quality control. For that, we use the maximum of the Brγ line and divide it by the noise level of the spectrum as indicated by the dashed fit displayed in the periodic pattern of the binary (Tables 7-9). The final SNR as a function of the epoch is shown in Fig. 4, where the threshold of 1 represents the noise level (black dashed line). For these data points, find an average SNR of 4.49 ± 2.27 where the uncertainty represents the standard deviation. While a threshold of SNR=2 is considered to be a non-artificial noise fluctuation, it is important to note that these measurements are executed on the reduced and sky-subtracted data cubes. It is well known that the dark and sky subtraction negatively impacts the SNR of the reduced data [24], which is why we conclude that all the data points used are of a physical nature. This argument

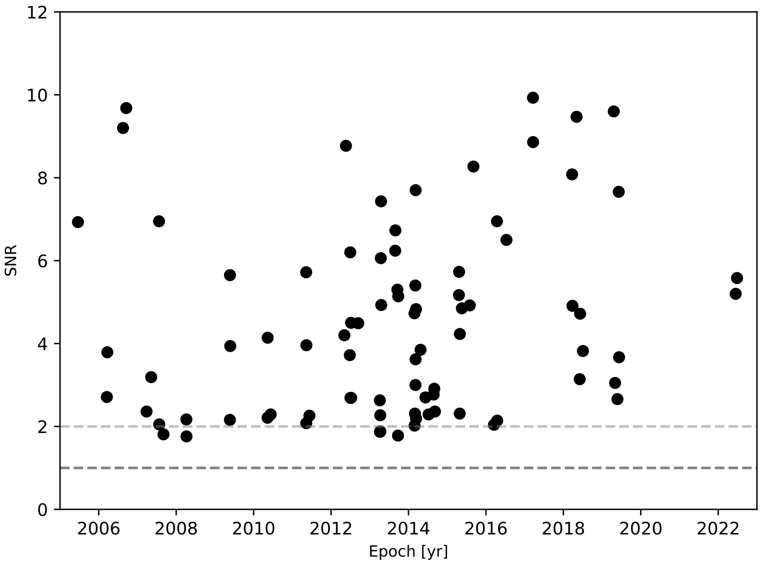

Supplementary Figure 4 Quality of the data investigated in this work. Except for five data points, all observations show a SNR above two (indicated with a gray dashed line).

is strengthened by the uniqueness of the IFU data points, i.e., all emission peaks show a line map counterpart. In order to visualize the relationship between the line maps and the Doppler-shifted Brγ emission peaks, we picked five observations with an SNR below two (Fig. 4). We display the related line maps together with the corresponding Brγ emission with the lowest SNR in the investigated data set in Fig. 5. Despite the low data quality, we detect D9 at the expected astrometric positions with an RV that matches the binary period presented in this work. The line maps in Fig. 5 exhibit emission above the noise and are similar to the detection of D9 in the high SNR data.

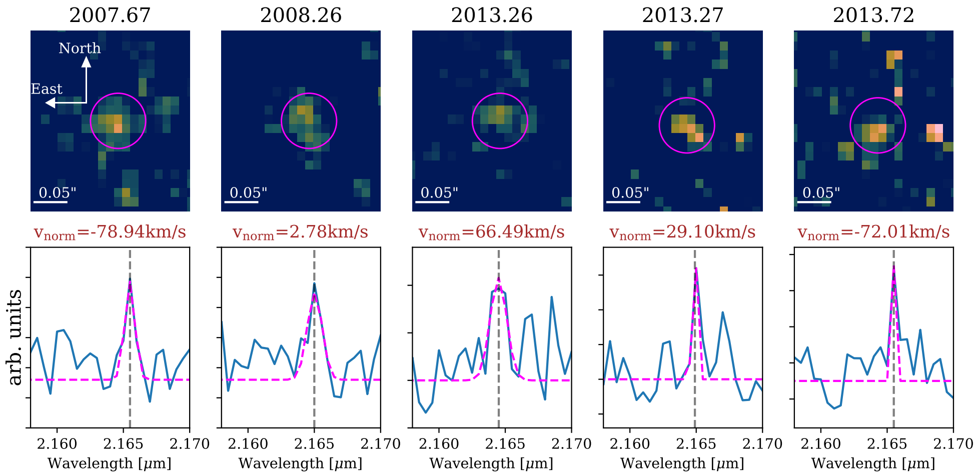

Supplementary Figure 5 Detection of the binary system D9 in the data set suffering from a low SNR. The selected data in this figure exhibit the highest noise level compared to any other data displayed in this work. However, the Brγ line is Doppler-shifted, which is why the emission of D9 can be distinguished from the sporadic noise in the data. D9 is in the center of each plot, north is up, east is to the left.

Supplementary Tables

ID	Period [days]	Reference
S2-36	39.42	[25]
S4-258 (E60)	2.30	[26]
S4-308	1.33	[8]
IRS 16SW	19.45	[6]
IRS 16NE	224.0	[26]
D9	372.3	This work

Supplementary Table 3 Binaries of the Galactic center. In this list, D9 is the only S cluster member.

Supplementary Note 1

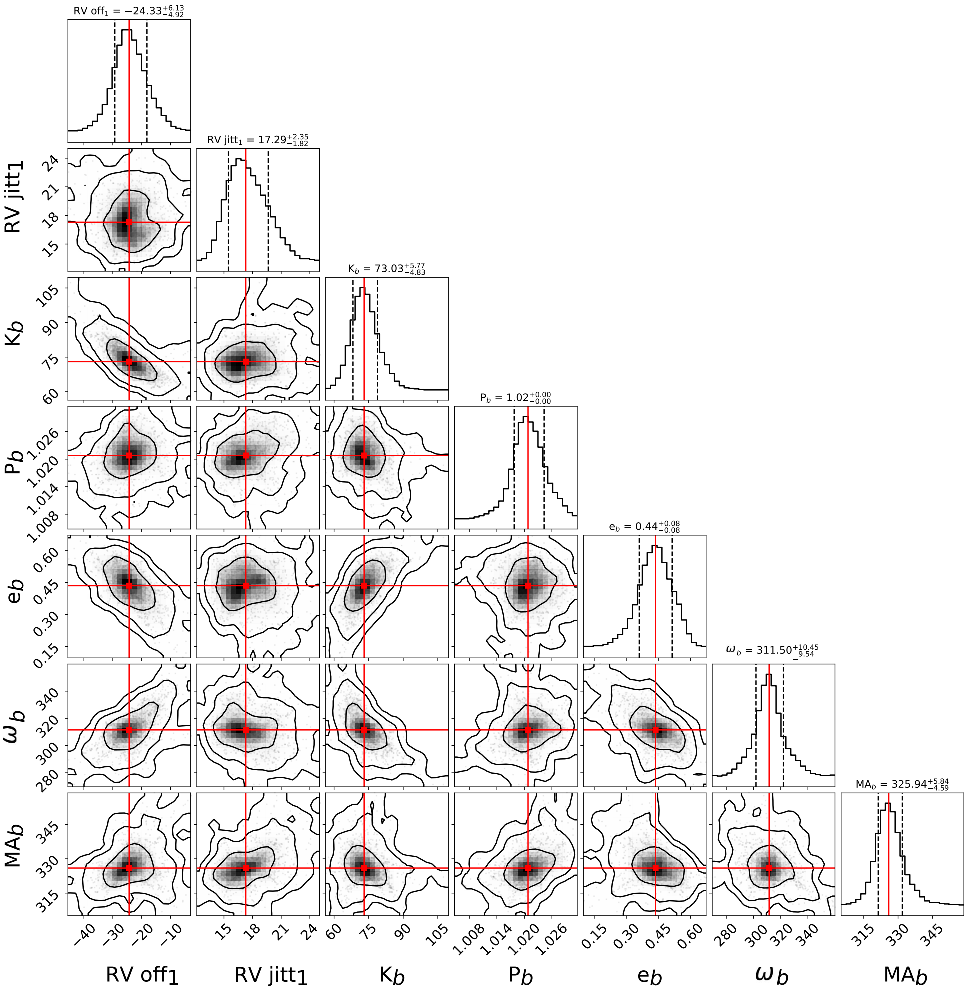

Supplementary Figure 6 Cornerplot of the Keplerian orbital parameters describing the secondary of the D9 binary system. We use the fit results of Exo-Stricker as priors to the MCMC model. The model is in high agreement with the fit results.

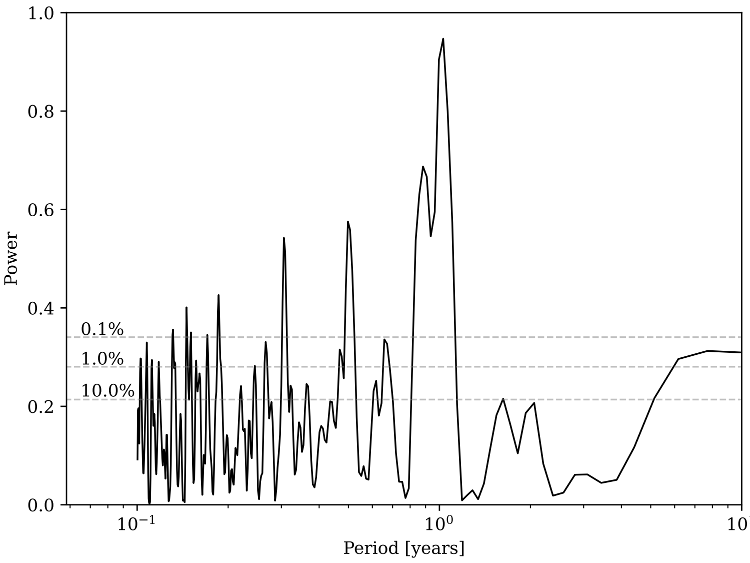

Supplementary Figure 7 Generalized Lomb-Scargle diagram of the frequency of the period. The x-axis is logarithmically scaled. The dashed lines indicate the False Alarm Probability (FAP) with 10.0%, 1.0%, and 0.1%. All peaks above are statistically significant. The peak maximum shows the LS power of 0.94 and can be found at 1.02 years.

Epoch	Wavelength [μm]	FWHM [μm]	RV [km/s]	ΔRV [km/s]	v_{norm} [km/s]	v_{mod} [km/s]
2005.463	2.16522	0.00035	-121.87	2.76	-24.92	4.27
2006.210	2.16498	0.00038	-155.11	1.38	8.30	37.49
2006.221	2.16499	0.00089	-153.73	8.30	6.92	36.11
2006.627	2.16549	0.00080	-84.48	1.38	-62.32	-33.12
2006.710	2.16546	0.00075	-88.63	2.76	-58.16	-28.96
2007.235	2.16496	0.00142	-157.88	9.69	11.07	40.26
2007.355	2.16501	0.00091	-150.96	4.15	4.15	33.34
2007.557	2.16549	0.00088	-84.48	2.76	-62.32	-33.12
2007.562	2.16548	0.00089	-85.86	5.53	-60.93	-31.74
2007.672	2.16561	0.00073	-67.86	11.07	-78.94	-49.75
2008.263	2.16489	0.00091	-167.58	9.69	20.77	49.96
2008.266	2.16500	0.00098	-152.34	1.38	5.53	34.72
2009.388	2.16541	0.00104	-95.56	5.53	-51.24	-22.05
2009.390	2.16548	0.00103	-85.86	2.76	-60.93	-31.74
2009.396	2.16539	0.00062	-98.33	9.69	-48.47	-19.27
2010.360	2.16487	0.00128	-170.35	9.69	23.54	52.73
2010.363	2.16489	0.00076	-167.58	11.07	20.77	49.96
2010.441	2.16500	0.00058	-152.34	1.38	5.53	34.72
2011.357	2.16498	0.00142	-155.11	2.76	8.30	37.49
2011.360	2.16501	0.00096	-150.96	4.15	4.15	33.34
2011.363	2.16501	0.00092	-150.96	1.38	4.15	33.34
2011.441	2.16497	0.00091	-156.50	4.15	9.69	38.88
2012.344	2.16458	0.00076	-210.51	6.92	63.70	92.89
2012.385	2.16498	0.00091	-155.11	2.76	8.30	37.49
2012.485	2.16502	0.00060	-149.57	2.76	2.76	31.95
2012.496	2.16499	0.00089	-153.73	5.53	6.92	36.11
2012.500	2.16501	0.00091	-150.96	1.38	4.15	33.34
2012.516	2.16500	0.00065	-152.34	1.38	5.53	34.72
2012.519	2.16551	0.00087	-81.71	4.15	-65.09	-35.90
2012.702	2.16550	0.00093	-83.09	1.38	-63.70	-34.51

Supplementary Table 4 Radial velocities measured from the SINFONI observations carried out between 2005-2012. We list the epoch, the wavelength, the corresponding Full-Width-Half-Maximum, the LOS velocity, and the related uncertainty. Furthermore, we indicate the normalized RV v_{norm} and the final velocity v_{mod} where the Keplerian model for the secondary from Exo-Stricker is included. All velocities, except v_{mod}, are estimated with respect to the Brγ rest wavelength at 2.1661 μm.

Epoch	Wavelength [μm]	FWHM [μm]	RV [km/s]	ΔRV [km/s]	v_{norm} [km/s]	v_{mod} [km/s]
2013.260	2.16451	0.00125	-220.21	5.53	66.47	95.66
2013.263	2.16477	0.00113	-184.20	6.92	30.46	59.65
2013.271	2.16478	0.00080	-182.81	1.38	29.08	58.26
2013.274	2.16502	0.00070	-149.57	2.76	-4.15	25.04
2013.285	2.16492	0.00108	-163.42	4.25	9.69	38.88
2013.291	2.16496	0.00116	-157.88	6.92	4.15	33.34
2013.296	2.16497	0.00076	-156.50	1.38	2.76	31.95
2013.657	2.16550	0.00090	-83.09	4.15	-70.63	-41.44
2013.662	2.16550	0.00070	-83.09	1.38	-70.63	-41.44
2013.713	2.16548	0.00105	-85.86	2.76	-67.86	-38.67
2013.729	2.16551	0.00072	-81.71	1.38	-72.01	-42.82
2013.735	2.16548	0.00099	-85.86	1.38	-67.86	-38.67
2014.154	2.16448	0.00117	-224.36	2.76	70.63	99.82
2014.157	2.16448	0.00122	-224.36	2.76	70.63	99.82
2014.166	2.16450	0.00100	-221.59	1.38	67.86	97.05
2014.177	2.16452	0.00078	-218.82	6.92	65.09	94.28
2014.180	2.16450	0.00089	-221.59	2.76	67.86	97.05
2014.183	2.16452	0.00084	-218.82	1.38	65.09	94.28
2014.185	2.16450	0.00081	-221.59	2.76	67.86	97.05
2014.194	2.16452	0.00075	-218.82	2.76	65.09	94.28
2014.196	2.16450	0.00073	-221.59	1.38	67.86	97.05
2014.312	2.16477	0.00127	-184.20	6.92	30.46	59.65
2014.438	2.16497	0.00118	-156.50	1.38	2.76	31.95
2014.519	2.16507	0.00104	-142.65	2.76	-11.07	18.12
2014.651	2.16543	0.00110	-92.79	8.30	-60.93	-31.74
2014.665	2.16550	0.00126	-83.098	1.38	-70.63	-41.44
2014.683	2.16555	0.00107	-76.173	1.38	-77.54	-48.35

Supplementary Table 5 Radial velocities measured from the SINFONI observations carried out between 2013-2014. Like Table 4, we list v_{mod} which is the final output velocity from the RV fitter Exo-Stricker.

Epoch	Wavelength [μm]	FWHM [μm]	RV [km/s]	ΔRV [km/s]	v_{norm} [km/s]	v_{mod} [km/s]
2015.302	2.16447	0.00108	-225.75	4.15	72.01	101.20
2015.307	2.16451	0.00088	-220.21	1.38	66.47	95.66
2015.323	2.16452	0.00109	-218.82	2.76	65.09	94.28
2015.326	2.16498	0.00132	-155.11	4.15	1.38	30.57
2015.379	2.16504	0.00106	-146.80	5.53	-6.92	22.27
2015.583	2.16513	0.00119	-134.34	6.92	-19.38	9.81
2015.677	2.16547	0.00075	-87.25	4.15	-66.47	-37.28
2016.207	2.16450	0.00099	-221.59	1.38	67.86	97.05
2016.285	2.16448	0.00090	-224.36	1.38	70.63	99.82
2016.291	2.16453	0.00094	-217.44	2.76	63.70	92.89
2016.530	2.16522	0.00111	-121.87	8.30	-31.85	-2.66
2017.215	2.16449	0.00114	-222.98	4.15	69.24	98.42
2017.218	2.16450	0.00075	-221.59	1.38	67.86	97.05
2018.226	2.16449	0.00075	-222.98	4.15	69.24	98.42
2018.235	2.16452	0.00077	-218.82	1.38	65.09	94.28
2018.341	2.16453	0.00134	-217.44	4.15	63.70	92.89
2018.422	2.16498	0.00094	-155.11	1.38	1.38	30.57
2018.433	2.16501	0.00130	-150.96	2.76	-2.76	26.43
2018.505	2.16502	0.00074	-149.57	1.38	-4.15	25.04
2019.302	2.16450	0.00105	-221.59	1.38	67.86	97.05
2019.336	2.16452	0.00091	-218.82	2.76	65.09	94.28
2019.396	2.16477	0.00107	-184.20	6.92	30.46	59.65
2019.430	2.16499	0.00114	-153.73	2.76	0.00	29.19
2019.438	2.16505	0.00141	-145.42	1.38	-8.30	20.89
2022.450	2.16446	0.00081	-227.14	1.38	73.42	102.61
2022.485	2.16458	0.00094	-210.51	1.38	56.79	85.98

Supplementary Table 6 Radial velocities measured from the SINFONI and ERIS observations carried out between 2015-2022. Like Table 4 and Table 5, we indicate all relevant observables that are used to derive the best-fit RV model displayed in the periodic pattern of D9. Here, v_{mod} indicates the final RV velocity. The data points from 2022 were observed with ERIS.

Epoch	Observation ID	Data quality			Exp. Time [s]	SNR [a.u.]		
		Total	Medium	Good		Noise	Peak	Ratio
2005.463	075.B-0547(B)	21	2	19	60	0.32	2.22	6.93
2006.210	076.B-0259(B)	5	0	3	600	0.84	2.28	2.71
2006.221	076.B-0259(B)	2	2	0	600	0.54	2.05	3.79
2006.627	077.B-0503(C)	5	0	5	600	0.39	3.58	9.20
2006.710	077.B-0503(C)	3	0	3	600	0.35	3.39	9.68
2007.235	078.B-0520(A)	8	1	2	600	1.32	3.12	2.36
2007.355	179.B-0261(F)	10	0	0	600	0.89	2.84	3.19
2007.557	179.B-0261(F)	3	0	2	600	0.23	1.60	6.95
2007.562	179.B-0261(Z)	7	0	7	600	1.72	3.54	2.05
2007.672	179.B-0261(K)	11	1	5	600	3.01	5.47	1.81
2008.263	081.B-0568(A)	16	0	15	600	1.29	2.80	2.17
2008.266	081.B-0568(A)	4	0	4	600	2.50	4.40	1.76
2009.388	183.B-0100(B)	7	0	7	600	0.65	1.41	2.16
2009.390	183.B-0100(B)	4	0	4	400	0.38	2.15	5.65
2009.396	183.B-0100(B)	3	0	3	600	0.59	2.33	3.94
2010.360	183.B-0100(O)	3	0	3	600	0.69	1.53	2.21
2010.363	183.B-0100(O)	5	0	5	600	0.35	1.45	4.14
2010.441	183.B-0100(O)	13	0	13	600	1.91	4.38	2.29
2011.357	087.B-0117(I)	3	0	3	600	0.33	1.89	5.72
2011.360	087.B-0117(I)	10	1	9	600	1.29	2.69	2.08
2011.363	087.B-0117(I)	6	0	6	600	0.57	2.26	3.96
2011.441	087.B-0117(I)	2	0	2	600	1.34	3.04	2.26
2012.344	288.B-5040(A)	2	0	2	600	0.60	2.52	4.20
2012.385	087.B-0117(J)	3	0	3	600	0.22	1.93	8.77
2012.485	087.B-0117(J)	1	0	1	600	0.86	3.20	3.72
2012.496	288.B-5040(A)	12	0	10	600	0.63	3.91	6.20
2012.500	288.B-5040(A)	4	0	4	600	1.73	4.67	2.69
2012.516	288.B-5040(A)	13	3	8	600	0.30	1.35	4.50
2012.519	087.B-0117(J)	2	1	1	600	0.66	1.78	2.69
2012.702	087.B-0117(J)	2	0	2	600	0.53	2.38	4.49

Supplementary Table 7 Analyzed SINFONI data of 2005 to 2012.

Epoch	Observation ID	Data quality			Exp. Time [s]	SNR [a.u.]		
		Total	Medium	Good		Noise	Peak	Ratio
2013.260	091.B-0088(A)	2	0	2	600	1.34	3.53	2.63
2013.263	091.B-0088(A)	8	0	8	600	1.39	2.60	1.87
2013.271	091.B-0088(A)	16	0	11	600	0.79	1.80	2.27
2013.274	091.B-0088(A)	3	0	3	600	2.17	4.09	1.88
2013.285	091.B-0088(A)	8	1	7	600	0.31	1.88	6.06
2013.291	091.B-0088(A)	3	0	3	600	0.30	2.23	7.43
2013.296	091.B-0088(A)	24	0	24	600	0.76	3.75	4.93
2013.657	091.B-0088(B)	10	1	6	600	0.25	1.56	6.24
2013.662	091.B-0088(B)	7	2	4	600	0.78	5.25	6.73
2013.713	091.B-0086(A)	6	0	6	600	0.10	0.53	5.30
2013.729	091.B-0086(A)	2	1	0	600	3.25	5.79	1.78
2013.735	091.B-0086(A)	3	1	1	600	0.54	2.78	5.14
2014.154	092.B-0920(A)	4	1	3	600	0.30	1.42	4.73
2014.157	091.B-0183(H)	7	3	1	400	0.85	1.72	2.02
2014.166	091.B-0183(H)	11	2	4	400	1.32	3.06	2.31
2014.177	091.B-0183(H)	3	0	3	400	0.37	2.00	5.40
2014.180	091.B-0183(H)	3	0	3	400	1.03	3.10	3.00
2014.183	091.B-0183(H)	3	0	3	400	0.29	1.05	3.62
2014.185	091.B-0183(H)	3	0	3	400	0.27	2.08	7.70
2014.194	092.B-0920(A)	6	0	6	400	0.24	1.16	4.83
2014.196	092.B-0920(A)	10	0	10	400	0.50	5.73	2.19
2014.312	092.B-0009(C)	13	0	13	400	0.49	1.89	3.85
2014.438	092.B-0009(C)	20	0	12	400	0.47	1.27	2.70
2014.519	093.B-0092(E)	14	3	0	400	0.72	1.65	2.29
2014.651	092.B-0398(A)	6	1	3	600	1.49	4.14	2.77
2014.665	093.B-0092(G)	4	3	0	400	0.36	1.05	2.91
2014.683	093.B-0218(B)	7	0	7	600	1.33	3.15	2.36

Supplementary Table 8 Analyzed SINFONI data of 2013 and 2014.

Epoch	Observation ID	Data quality			Exp. Time [s]	SNR [a.u.]		
		Total	Medium	Good		Noise	Peak	Ratio
2015.302	095.B-0036(A)	12	0	12	600	0.47	2.43	5.17
2015.307	095.B-0036(A)	9	0	9	600	0.38	2.18	5.73
2015.323	095.B-0036(A)	14	0	14	600	2.66	6.16	2.31
2015.326	095.B-0036(A)	12	0	8	600	0.97	4.11	4.23
2015.379	095.B-0036(A)	3	0	3	600	1.11	5.39	4.85
2015.583	095.B-0036(C)	23	7	8	400	0.78	3.84	4.92
2015.677	095.B-0036(D)	17	11	4	400	0.18	1.49	8.27
2016.207	096.B-0157(B)	17	0	17	400	0.66	1.35	2.04
2016.285	594.B-0498(R)	12	0	12	600	0.24	1.67	6.95
2016.291	594.B-0498(R)	10	0	8	600	1.57	3.36	2.14
2016.530	097.B-0050(A)	27	0	13	600	0.06	0.39	6.50
2017.215	598.B-0043(D)	11	0	5	600	0.16	1.59	9.93
2017.218	598.B-0043(D)	15	4	11	600	0.15	1.33	8.86
2018.226	598.B-0043(D)	8	0	8	600	0.12	0.97	8.08
2018.235	598.B-0043(D)	12	1	9	600	0.24	1.18	4.91
2018.341	598.B-0043(E)	17	0	17	600	0.17	1.61	9.47
2018.422	598.B-0043(F)	8	0	8	600	1.61	5.07	3.14
2018.433	598.B-0043(F)	14	1	7	600	0.25	1.18	4.72
2018.505	598.B-0043(G)	22	12	10	600	0.45	1.72	3.82
2019.302	0103.B-0026(B)	9	0	8	600	0.10	0.96	9.60
2019.336	0103.B-0026(F)	8	0	8	600	0.52	1.59	3.05
2019.396	5102.B-0086(Q)	4	0	2	600	0.89	2.37	2.66
2019.430	594.B-0498(Q)	11	0	10	600	0.24	1.84	7.66
2019.438	5102.B-0086(Q)	14	0	10	600	0.34	1.25	3.67
2022.450	60.A-9917(C)	3	0	2	600	20.1	104.56	5.20
2022.485	60.A-9917(D)	3	0	2	600	57.75	322.58	5.58

Supplementary Table 9 Analyzed SINFONI and ERIS data of 2015 to 2022. The two observations in 2022 were carried out with ERIS.

References

[1] Schödel, R. *et al.* A star in a 15.2-year orbit around the supermassive black hole at the centre of the Milky Way. *Nature* **419**, 694–696 (2002).

[2] Gillessen, S. *et al.* Monitoring Stellar Orbits Around the Massive Black Hole in the Galactic Center. *Astrophys. J.* **692**, 1075–1109 (2009).

[3] Peißker, F. *et al.* The Evaporating Massive Embedded Stellar Cluster IRS 13 Close to Sgr A*. I. Detection of a Rich Population of Dusty Objects in the IRS 13 Cluster. *Astrophys. J.* **956**, 70 (2023c).

[4] Eisenhauer, F. *et al.* SINFONI in the Galactic Center: Young Stars and Infrared Flares in the Central Light-Month. *Astrophys. J.* **628**, 246–259 (2005).

[5] Ali, B. *et al.* Kinematic Structure of the Galactic Center S Cluster. *Astrophys. J.* **896**, 100 (2020).

[6] Ott, T., Eckart, A. & Genzel, R. Variable and Embedded Stars in the Galactic Center. *Astrophys. J.* **523**, 248–264 (1999).

[7] Viehmann, T., Eckart, A., Schödel, R., Pott, J. U. & Moultaka, J. Dusty Sources at the Galactic Center the N- and Q-Band Views with VISIR. *Astrophys. J.* **642**, 861–867 (2006).

[8] Gautam, A. K. *et al.* An Estimate of the Binary Star Fraction Among Young Stars at the Galactic Center: Possible Evidence of a Radial Dependence. *arXiv e-prints* arXiv:2401.12555 (2024).

[9] Peißker, F. *et al.* Candidate young stellar objects in the S-cluster: Kinematic analysis of a subpopulation of the low-mass G objects close to Sgr A*. *Astron. Astrophys.* **686**, A235 (2024).

[10] Labadie, L. *et al.* High-contrast optical imaging of companions: the case of the brown dwarf binary HD 130948 BC. *Astron. Astrophys.* **526**, A144 (2011).

[11] Habibi, M. *et al.* Twelve Years of Spectroscopic Monitoring in the Galactic Center: The Closest Look at S-stars near the Black Hole. *Astrophys. J.* **847**, 120 (2017).

[12] Schödel, R., Najarro, F., Muzic, K. & Eckart, A. Peering through the veil: near-infrared photometry and extinction for the Galactic nuclear star cluster. Accurate near infrared H, Ks, and L' photometry and the near-infrared extinction-law toward the central parsec of the Galaxy. *Astron. Astrophys.* **511**, A18 (2010).

[13] Sabha, N. *et al.* The S-star cluster at the center of the Milky Way. On the nature of diffuse NIR emission in the inner tenth of a parsec. *Astron. Astrophys.* **545**, A70 (2012).

[14] Fritz, T. K. *et al.* Line Derived Infrared Extinction toward the Galactic Center. *ApJ* **737**, 73 (2011).

[15] Peißker, F. *et al.* X3: A High-mass Young Stellar Object Close to the Supermassive Black Hole Sgr A*. *Astrophys. J.* **944**, 231 (2023b).

[16] Whitney, B. A., Indebetouw, R., Bjorkman, J. E. & Wood, K. Two-Dimensional Radiative Transfer in Protostellar Envelopes. III. Effects of Stellar Temperature. *Astrophys. J.* **617**, 1177–1190 (2004).

[17] Keppler, M. *et al.* Discovery of a planetary-mass companion within the gap of the transition disk around PDS 70. *Astron. Astrophys.* **617**, A44 (2018).

[18] Garcia, P. J. V. *et al.* Pre-main-sequence binaries with tidally disrupted discs: the Brγ in HD 104237. *Mon. Not. R. Soc.* **430**, 1839–1853 (2013).

[19] Dinh, C. K. *et al.* High-resolution, Mid-infrared Color Temperature Mapping of the Central $10''$ of the Galaxy. *Astron. J.* **167**, 41 (2024).

[20] Shimizu, T., Uyama, T., Hori, Y., Tamura, M. & Wallack, N. High-contrast Imaging around a 2 Myr-old CI Tau with a Close-in Gas Giant. *Astron. J.* **165**, 20 (2023).

[21] Stapper, L. M., Hogerheijde, M. R., van Dishoeck, E. F. & Mentel, R. The mass and size of Herbig disks as seen by ALMA. *Astron. Astrophys.* **658**, A112 (2022).

[22] Peißker, F., Eckart, A., Sabha, N. B., Zajaček, M. & Bhat, H. Near- and Mid-infrared Observations in the Inner Tenth of a Parsec of the Galactic Center Detection of Proper Motion of a Filament Very Close to Sgr A*. *Astrophys. J.* **897**, 28 (2020c).

[23] Gravity Collaboration *et al.* Detection of the gravitational redshift in the orbit of the star S2 near the Galactic centre massive black hole. *Astron. Astrophys.* **615**, L15 (2018).

[24] Newberry, M. V. Signal-to-Noise Considerations for Sky-Subtracted CCD Data. *Publ. Astron. Soc. Pacif.* **103**, 122 (1991).

[25] Gautam, A. K. *et al.* An Adaptive Optics Survey of Stellar Variability at the Galactic Center. *Astrophys. J.* **871**, 103 (2019).

[26] Pfuhl, O. *et al.* Massive Binaries in the Vicinity of Sgr A*. *Astrophys. J.* **782**, 101 (2014).
